# Motivation to smoking cessation in head and neck cancer and dysplasia patients in confrontation with the attitudes of otorhinolaryngologists in delivering anti-smoking therapies

**DOI:** 10.1007/s00405-021-07209-2

**Published:** 2021-12-10

**Authors:** Anna Rzepakowska, Bartosz Marcinkiewicz, Michał Żurek, Dominika Wiśniewska, Kazimierz Niemczyk

**Affiliations:** 1 Department of Otorhinolaryngology Head and Neck Surgery, Medical University of Warsaw, ul. Banacha 1a, 02-097 Warszawa, Poland; 2 Students Scientific Research Group at the Department of Otorhinolaryngology Head and Neck Surgery, Medical University of Warsaw, Warsaw, Poland; 3 Doctoral School, Medical University of Warsaw, Warsaw, Poland; 4 Institute of Psychology, The Maria Grzegorzewska University, Warsaw, Poland

**Keywords:** Oral cavity cancer, Oropharyngeal cancer, Laryngeal cancer, Hypopharyngeal cancer, Laryngeal dysplasia, Smoking cessation, Anti-smoking therapy

## Abstract

**Objectives:**

The aim of the study was to determine the influence of oral, oropharyngeal, laryngeal and hypopharyngeal dysplasia and cancer diagnosis on motivation to smoking cessation in patients. Consecutively, we assessed the competence of ENT specialists in counseling anti-smoking therapies.

**Methods:**

Questionnaire of expected support, Schneider motivation test and Fagerström Test for Nicotine Dependence (FTND) were administered to 50 smoking patients. The online survey was collected from 152 ENT doctors.

**Results:**

Mean FTND score was 4.58 and Heaviness of Smoking Index (HSI) was 3.1. Patients with oral cavity and oropharyngeal cancer showed the greatest dependence to nicotine 7.67 and 5.25, respectively, and with hypopharyngeal cancer had the lowest 3.5, (*p* = 0.039). The ranges of HSI were significantly higher for younger patients (*p* = 0.036). 35 patients were adequately motivated to quit smoking, and their mean age was statistically higher (*p* = 0.05). Self-reported motivation to smoking cessation was 76%. Of 152 surveyed doctors, only 39% declared knowledge of the diagnostic and therapeutic cessation interventions. 75% showed interest in the training programs.

## Introduction

Smoking is a major etiological factor for developing intraepithelial dysplasia and squamous cell carcinomas (SCC) of the larynx, hypopharynx, oropharynx and oral cavity. Despite increased incidence of human papilloma virus related SCC in oropharynx and oral cavity, still more than 75% head and neck cancers are attributed to tobacco exposure with or without alcohol consumption [1].

According to the report of the Chief Sanitary Inspector “Attitudes of Poles towards tobacco smoking”, 21% of adult population in Poland reported smoking addiction in 2019. In recent years, there has been observed (a downward trend in the prevalence of smoking) a gradual decline in the percentage of smokers and the difference compared to the results from 2011 was 10 percentage points (21% compared to 31%). The largest group among smokers are men in their 5th decade of life [2].

In 2017, the percentage rate of laryngeal cancer incidence and mortality among all malignancies in Poland was, respectively, 2.3% and 2.5% [3]. Cancers of other localizations within upper digestive tract showed a 3.5% share among all malignancies’ incidence [3]. Worldwide data confirm high proportion of current smokers among diagnosed head and neck cancers with reported range from 41 to 56% [4, 5]. Even more concerning are the rates of continued smoking despite the diagnosis of cancer, and they exceed 50% of patients [6]. It has been already proved that continued smoking contributes to increased risk of intraoperative and postoperative complications, poor treatment response, and treatment-related toxic effects [7]. Moreover, it has negative impact on disease-free survival and overall survival and is associated with increase in the rates of second primaries [7]. Evidence suggests that quitting smoking at the time of cancer diagnosis can decrease the death risk even by 30–40% [7, 8] and it results in further improvement of psychological functioning and live quality of cancer patients [9].

Current recommendations offer head and neck cancer patients comprehensive methods for diagnosis, treatment and follow-up; however, tobacco assessment and treatment practices are still not commonly included in the oncologic setting. Despite the consensus on the importance of smoking cessation, even half of surgeons do not counsel their patients [10]. The clinical targeting on smoking population with cancer for comprehensive tobacco intervention including motivational interviewing and nicotine replacement therapy or pharmacotherapy is still undervalued and not meticulously organized by many cancer centers. Although the wish to stop smoking is expressed by more than 60% of smokers, the estimations shows that only about 2% of them quit smoking annually without any help, and further 2–19% will succeed depending on the quality of the professional support [11].

Cancer suspicious or diagnosis may contribute to patient’s decision to quit smoking and make them more receptive to cessation treatment [12, 13]. These diagnoses personalize harms of tobacco and concentrate patients’ priorities on restoration and maintenance of good health. However, the severe nicotine dependence, cancer-related distress and depression may reduce the benefit of motivating moment of the diagnosis if the patient is not treated properly. Therefore, the question raises about the specialist, who should deliver the cessation intervention. Do head and neck surgeons feel adequately prepared to assist smoking cessation in their cancer patients? Is it realistic to demand from surgeons managing the cancer treatment and follow-up process as well as the time-consuming tobacco treatment in the oncologic setting?

The need for implementation and enhancement of smoking cessation therapy in oncological practice is worldwide recognized and agreed issue in the field [14]. The adequate and optimal solutions must be developed to improve tobacco cessation capacity.

Primary objective of the study is to evaluate the effect of dysplasia or head and neck cancer diagnosis on interest in cessation programs among surgical patients and assess their preferences for such intervention including character of smoking, attitudes toward the coordinating specialist and nicotine replacement therapy and/or pharmacotherapy. An additional objective was the evaluation of knowledge and attitudes toward delivering anti-smoking therapies by otorhinolaryngologists.

## Materials and methods

### Participants

Smoking patients presenting for a surgical treatment at our center were invited into the study. Eligible participants were at least 18 years of age, with dysplasia or squamous cell cancer of oral cavity, oropharynx, larynx or hypopharynx, capable of providing informed consent, able to speak. Patients cognitively impaired and with cancer stage for palliative treatment were not eligible.

The additional study group was otorhinolaryngology specialists or doctors during the specialization training who were addressed online.

### Procedures and measures

#### Patient participants completed

-A short survey to assess sociodemographic (age, gender, employment status), duration of ENT disease symptoms, current health status and basic smoking characteristics (duration, cigarettes per day, quit attempts, and interest in cessation interventions in connection with diagnosis suspicious of cancer).

-The interviewer-administered questionnaire evaluating preferences for smoking cessation strategies (characteristics of previous cessation attempts, experiences with qualified persons, expected support including an indication of preferred specialist, attitudes toward replacement therapy, and main source of information on quitting).

-Fagerström Test for Nicotine Dependence (FTND) [15]—a validated standardized 6-item test for assessment of the intensity of physical addiction to nicotine related to cigarette smoking. It evaluates the quantity of cigarette consumption, the compulsion to use and dependence. In scoring the FTND, three “yes/no” items are scored from 0 to 1 and three multiple choice items are scored from 0 to 3. The items are summed to yield a total score of 0–10. The higher the total FTND score, the more intense is the patient’s physical dependence on nicotine. Classification of dependence: 0–2—very low, 3–4—low, 5—moderate, 6–7—high, 8–10—very high.

-Test of Motivation for Ceasing Smoking by Nina Schneider [16]—a standard instrument containing 12 questions for which you can answer “yes” or “no” with a 2-point score (0–1). Test result was considered a low motivation below 7 points, and a score equal or higher than 7 points for high motivation.

The tumor site and histopathological diagnosis were abstracted from medical documentation.

ENT specialists and doctors in residency completed online survey with 17 questions (14 closed and 3 open questions). First 5 questions were related to seniority, place of work, gender and smoking habit of the doctor, next 12 considered the experience with tobacco counseling, performing assessment of motivation to quit and smoking dependence, methods of used therapies in smoking cessation, interest in delivering comprehensive cessation treatment, interest in training courses about strategies in smoking cessation intervention.

### Statistical analyses

Statistical analysis was performed using Microsoft Excel 2016 and IBM^®^ SPSS^®^ Statistics 25. *P* values below 0.05 were considered statistically significant. Descriptive statistics and frequencies presented the study variables. The Fagerström Test for Nicotine Dependence (FTND) scores were calculated by summing responses to the 6 FTND questions and using the response scale provided with the test. The Heaviness of Smoking Index was calculated by summing the responses to FTND item 1 and FTND item 4, using the response scale. The time to first cigarette (TTFC) metric was calculated from FTND item 1 and recoded into two groups: 31 or more min, and 30 min or less. Mann–Whitney *U* tests and one-way ANOVA on ranks were used to evaluate continuous variables measured on the ratio scale. In the case of the one-way ANOVA on ranks, statistically significant results were confirmed using post hoc tests (Dunn tests with Bonferroni correction). The analysis of nominal variables was based on the construction of cross tables and the Chi-square test of independence. In the case of variables with an expected distribution lower than 5, the Yates continuity correction was applied. Each answer to multiple choice questions was analyzed separately, and the results of the Chi-square tests of independence for each answer apply to the combined remaining responses.

## Results

### Smoking patients’ results

Of the 50 patients (42 men, 8 women), 21 (42%) had diagnosis of laryngeal cancer, 14 (28%)—laryngeal dysplasia, 8 (16%)—oropharyngeal cancer, 4 (8%)—hypopharyngeal cancer, and 3 (6%)—cancer of oral cavity. Mean age was 63.02 ± 7.86 years and there were found no significant age differences for sex and type of disease. Professional activity was declared by 23 (46%) patients, 21 (42%) retired, and 6 (12%) were unemployed. The most common reported comorbidity was arterial hypertension—24 patients. The mean smoking period among all patients was 38.8 years. The mean nicotine dependency ratio based on the Fagerström test was 4.58 ± 2.23; the mean value of the Heaviness of Smoking Index (HSI) was 3.1 ± 1.73. 22 patients (50%) smoked 10–20 cigarettes a day, 11 (22%)—21–30 cigarettes a day, 8 (16%)—less than 10, and 6 (12%) more than 30 cigarettes a day (Table 1).

**Table 1:** Smoking status in patients with dysplasia and cancer

Variable	*n* (%)	Age (years); Mean ± SD	*p* value	Duration smoked (years); Mean ± SD	*p* value	Nicotine dependence (score); Mean ± SD	*p* value	Heaviness of Smoking Index (score); Mean ± SD	*p* value
All patients	50 (100)	63.02 ± 7.86		38.78 ± 8.44		4.58 ± 2.23		3.1 ± 1.73	
Sex									
Men	42 (84)	63.19 ± 7.62	0.866^a^	39.62 ± 7.7	0.266^a^	4.5 ± 2.28	0.524^a^	3.02 ± 1.76	0.412^a^
Women	8 (16)	62.13 ± 9.55		34.38 ± 11.16		5 ± 2.07		3.5 ± 1.6	
Type of disease									
Dysplasia	14 (28)	62.14 ± 6.85	0.066^b^	37.79 ± 9.31	0.268^b^	3.64 ± 2.06	**0.039**^**b**^	2.5 ± 1.91	0.093^b^
Oral cavity cancer	3 (6)	49.67 ± 6.66		28.33 ± 7.64		7.67 ± 0.58		5.33 ± 0.58	
Oropharynx cancer	8 (16)	61.25 ± 9.13		39.13 ± 8.31		5.25 ± 1.16		3.25 ± 1.04	
cancer	21 (42)	65.71 ± 6.92		41.10 ± 7.71		4.74 ± 2.43		1.81	
Hypopharynx cancer	4 (8)	65.5 ± 3.87		37.72 ± 6.08		3.5 ± 1.91		2.5 ± 1	
Employment status									
Employed	23 (46)	58.35 ± 6.88	**<0.001**^**b**^	34.7 ± 7.45	**0.004**^**b**^	5.21 ± 2.21	0.098^b^	3.61 ± 1.62	0.138^b^
Retired	21 (42)	68.67 ± 5.77		43.14 ± 8.25		3.81 ± 2.04		1.72	
Unemployed	6 (12)	61.17 ± 5.04		39.17 ± 4.92		4.83 ± 2.48		3 ± 1.9	
Smoking per day									
≤10 Cigarettes	8 (16)	68.88 ± 5.84	0.061^b^	40.75 ± 8.88	0.900^b^	1.5 ± 0.93	**<0.001**^**b**^	0.63 ± 0.74	**<0.001**^**b**^
11–20 Cigarettes	25 (50)	63.08 ± 8.13		38.92 ± 9.11		4.36 ± 1.68		2.84 ± 1.14	
21–30 Cigarettes	11 (22)	60.55 ± 8.15		38 ± 8.19		6.18 ± 1.17		4.45 ± 1.04	
≥31 Cigarettes	6 (12)	59.5 ± 4.93		37 ± 6.63		6.67 ± 2.25		5 ± 1.26	
Nicotine dependence score^b^									
Very low (0–2)	12 (24)	67.08 ± 6.91	0.091^b^	39.25 ± 8.92	0.570^b^			0.92 ± 0.79	**<0.001**^**b**^
Low (3–4)	9 (18)	63.78 ± 7.81		41.11 ± 9.94				2.11 ± 0.78	
Medium (5)	11 (22)	64.09 ± 6.25		40.73 ± 5.79				3.73 ± 0.65	
High (6–7)	13(26)	60.54 ± 8.12		36 ± 8.45				0.85	
Very high (8 to 10)	5 (10)	56 ± 8.52		36.4 ± 10.11				5.6 ± 0.55	
Heaviness of smoking index									
low (0-1)	11 (22)	66.91 ± 7.05	**0.036**^**b**^	38.73 ± 8.59	0.700^b^	1.64 ± 0.92	**<0.001**^**b**^		
medium (2-4)	28 (56)	63.32 ± 7.67		39.5 ± 8.82		1.38			
high (5-6)	11 (22)	58.36 ± 7.31		37 ± 7.75		7.18 ± 1.17			
Time to first cigarette									
≤30 Minutes	23 (46)	65.52 ± 7.45	**0.039**^**a**^	39.48 ± 8.87	0.865^a^	2.87 ± 1.63	**<0.001**^**a**^	1.7 ± 1.22	**<0.001**^**a**^
≥31 Minutes	27 (54)	60.89 ± 7.7		38.19 ± 8.18		6.04 ± 1.53		4.29 ± 1.07	
Motivated to quit smoking									
Yes	35 (70)	64.71 ± 6.73	0.051^a^	39.43 ± 8.14	0.403^a^	4.26 ± 2.19	0.140^a^	2.74 ± 1.72	%1.%2^**a**^
No	15 (30)	59.07 ± 9.01		37.27 ± 9.22		5.33 ± 2.25		3.93 ± 1.47	

Bold indicates *p* ≤ 0.05

^a^Mann–Whitney *U* test

^b^One-way ANOVA on ranks

^c^By the Fagerström test for nicotine dependence

Nicotine dependence ratio had significantly different values depending on the type of laryngological disease of the patients (*p* = 0.039). These with oral cavity and oropharyngeal cancer showed the greatest dependence to nicotine (7.67 ± 0.58 and 5.25 ± 1.16, respectively), and patients with hypopharyngeal cancer had the lowest (3.5 ± 1.91).

The duration of smoking was significantly correlated with the employment status (*p* = 0.004) and was the longest for retired and unemployed patients (43.14 ± 8.25 and 39.17 ± 4.92 years, respectively). The ranges of the Heaviness of Smoking Index differed significantly depending on the age of the patients (*p* = 0.036). On average, the younger patients (58.36 ± 7.31. 23) showed a high HSI (5–6 points), and those with the lowest smoking intensity—HSI (0–1 point) were, on average, the oldest (66.91 ± 7.05). 23 patients (46%) had time to first cigarette (TTFC) shorter than 31 min and they were statistically (*p* = 0.039) younger (65.52 ± 7.45) compering to these with TTFC longer than 30 min. Based on the results of the motivation test according to Schneider, 35 patients (70%) were adequately motivated to quit smoking and their mean age was statistically higher (*p* = 0.05) comparing to unmotivated patients (64.71 ± 6.73 and 59.07 ± 9.01, respectively). Patients motivated to quit smoking had statistically (p = 0.025) lower HSI (2.74 ± 1.72 and 3.93 ± 1.47, respectively). To the question “Can the diagnosis of upper aerodigestive tract affect your decision to stop smoking?”, 38 (76%) patients answered affirmatively, 7 (14%)—negative, and 5 (10%)—did not have an opinion. There were no significant differences in patients’ decisions depending on gender, location and type of the disease, and the severity of smoking dependence. 40 (80%) of the patients reported previous attempts to quit smoking. In addition, no significant relationships were found between attempts to quit depending on gender, location and type of the disease and the severity of smoking dependence (Table 2).

**Table 2:** Smoking cessation trends regarding cancer diagnosis and treatment

Variable	Motivated to quit smoking [*n* (%)]^a^	*p* value^b^	Declared previous attempts of quitting smoking [*n* (%)]	*p* value^b^	Duration of disease (months); Median ± IR	*p* value	Declared positive impact on quitting smoking due to disease [*n* (%)]	*p* value^b^
All patients	35 (70)		40 (80)		8 ± 8		38 (76)	
Sex								
Men	30 (71.14)	0.933	33 (78.57)	0.563	8 ± 8	0.765^c^	32 (76.19)	0.176
Women	5 (62.5%)		7 (87.5)		9 ± 36		6 (75)	
Type of disease								
Dysplasia	11 (78.57)	0.511	11 (78.57)	0.861	12 ± 16	0.103^d^	11 (78.57)	0.692
Oral cavity cancer	1 (33.33)		3 (100)		10		1 (33.33)	
Oropharynx cancer	6 (75)		7 (87.5)		4.5 ± 3		7 (87.5)	
Larynx cancer	15 (71.14)		16 (76.19)		9 ± 7		16 (76.19)	
Hypopharynx cancer	2 (50)		3 (75)		4.5 ± 3		3 (75)	
Smoking per day								
≤10 Cigarettes	7 (87.5)	0.188	6 (75)	0.854	7 ± 8	0.904^d^	8 (100)	0.095
11–20 Cigarettes	19 (76)		21 (84)		6 ± 9		20 (80)	
21–30 Cigarettes	5 (45.45)		8 (72.73)		12 ± 8		8 (72.73)	
≥31 Cigarettes	4 (66.67)		5 (83.33)		9 ± 12		2 (33.33)	
Nicotine dependence score								
Very low (0–2)	10 (83.33)	0.458	10 (83.33)	0.759	6 ± 8	0.783^d^	11 (91.67)	0.865
Low (3–4)	7 (77.77)		7 (77.77)		8 ± 21		7 (77.77)	
Medium (5)	7 (63.63)		10 (90.91)		6 ± 9		7 (63.63)	
High (6–7)	9 (69.23)		9 (69.23)		12 ± 21		10 (76.92)	
Very high (8 to 10)	2 (40)		4 (80)		10 ± 13		3 (60%)	
Heaviness of smoking index								
low (0-1)	10 (90.91)	0.065	10 (90.91)	0.254	6 ± 8	0.271^d^	11 (100)	0.292
medium (2-4)	20 (71.14)		23 (82.14)		6 ± 8		20 (71.14)	
high (5-6)	5 (45.45)		7 (63.64)		12 ± 18		7 (63.64)	
Time to first cigarette								
≤30 Minutes	18 (78.26)	0.386	21 (91.3)	0.136	6 ± 8	0.085^c^	19 (82.61)	0.556
≥31 Minutes	17 (62.96)		19 (70.37)		12 ± 20		19 (62.96)	

Bold indicates *p* ≤ 0.05

^a^By the Schneider Motivation Test

^b^For Chi-square test of independence

^c^Mann–Whitney *U* test

^d^One-way ANOVA on ranks

Only 6 (12%) of the surveyed patients used the help of qualified personnel on smoking cessation attempts in the past, and 22 patients (44%) declared the need for support when trying to quit smoking. An open-ended question with the possibility of multiple choice: “Whose support in quitting smoking will be the most important for you”, 16 patients indicated family members, 12—a psychologist, 10—an ENT specialist, 8—an anti-smoking professional, and 6—a family doctor. Among the various professions who should deliver smoking cessation therapy, 14 patients indicated a psychologist, 11—a family doctor, 11—an ENT doctor, 8—an anti-smoking professional, and 3—a pulmonologist. 33 patients (66%) believed that smoking should be treated as a disease and patients with a lower nicotine dependence index had such an opinion significantly more often (*p* = 0.019). Thirty patients (66%) declared that they would accept pharmacological treatment when trying to quit smoking (Table 3).

**Table 3:** Interest in smoking cessation among patients with dysplasia and cancer

Variable	Declared professional smoking-cessation advice [*n* (%)]		*p* value^a^	Declared need of support in quitting smoking [*n* (%)]	*p* value^a^	Declared smoking as disease [*n* (%)]	*p* value^a^	Declared acceptance of pharmacotherapy [*n* (%)]	*p* value^a^
All patients	6 (12)			22 (44)		33 (66)		30 (60)	
Sex									
Men	4 (9.52)		0.217	16 (38.1)	0.054	26 (61.9)	0.161	23 (54.76)	0.083
Women	2 (25)			6 (75)		7 (87.5)		7 (87.5)	
Type of disease									
Dysplasia	2 (14.29)		0.522	6 (42.86)	0.763	12 (85.71)	0.068	9 (64.86)	0.981
Oral cavity cancer	1 (33.33)			1 (33.33)		0 (0)		2 (66.67)	
Oropharynx cancer	0 (0)			5 (62.5)		5 (62.5)		5 (62.5)	
Larynx cancer	2 (9.52)			9 (42.86)		14 (66.67)		12 (57.14)	
Hypopharynx cancer	1 (25)			1 (25)		2 (50)		2 (50)	
Smoking per day:									
≤10 Cigarettes	2 (25)		0.407	3 (37.5)	0.455	6 (75)	0.06	3 (37.5)	0.09
11–20 Cigarettes	3 (12)			13 (52)		18 (72)		14 (56)	
21–30 Cigarettes	0 (0)			5 (45.45)		8 (72.72)		10 (90.91)	
≥31 Cigarettes	1 (16.67)			1 (16.67)		1 (16.67)		3 (50)	
Nicotine dependence score									
Very low (0–2)	3 (25)		0.172	3 (25)	0.101	11 (91.67)	**0.019**	6 (50)	0.539
Low (3–4)	2 (22.22)			7 (77.78)		6 (66.67)		7 (77.78)	
Medium (5)	0 (0)			6 (54.54)		9 (81.82)		5 (45.45)	
High (6–7)	0 (0)			5 (38.46)		6 (46.15)		9 (69.23)	
Very high (8 to 10)	1 (20)			1 (20)		1 (20)		3 (60)	
Heaviness of Smoking Index									
Low (0-1)	3 (27.27)		0.208	5 (45.45)	0.434	9 (81.82)	0.188	6 (54.54)	0.614
Medium (2-4)	2 (7.14)			14 (50)		19 (67.86)		16 (57.14)	
High (5-6)	1 (9.09)			3 (27.27)		5 (45.45)		8 (72.73)	
Time to first cigarette									
≤30 Minutes	5 (21.73)		**0.05**	12 (52.17)	0.283	18 (78.26)	0.091	16 (69.57)	0.203
≥31 Minutes	1 (3.7)			10 (37.03)		15 (55.56)		14 (50)	

Bold indicates *p* ≤ 0.05

^a^For Chi-square test of independence

### Surveyed doctors’ results

The online version of anonymous survey was mailed to 900 doctors professionally related to the otorhinolaryngology specialization. A total of 152 doctors (17%) sent their answers. Among the respondents, 68% (*n* = 103) were women and 32% were men (*n* = 49). The form used did not require answering all the questions, therefore, the number of answers for individual questions may vary. 113 (74%) applied doctors declared the title of specialist in otolaryngology. The work experience of 103 respondents (67%) exceeded 10 years. 149 people replied to the question about the type of performed work. The percentage of physicians who declared public outpatient consultations (67%; *n* = 102) was similar to both the percentage of people providing commercial outpatient consultations (68%; *n* = 104) and doctors performing surgical procedures (63%; *n* = 97). Smoking was declared by 7% of the surveyed doctors (*n* = 10). A higher percentage of smokers concerns doctors without specialization (17.95% vs. 2.65%) and these with work experience beneath 10 years (16 vs. 2%). The dependencies of smoking and specialist title and duration of work experience proved to be statistically significant (*p* = 0.003 and *p* = 0.004, respectively).

Most responders (65%, *n* = 99) declared that they always ask their patients about smoking on history. 41 doctors (27%) ask only if patients’ disease is related to smoking. 11 surveyed doctors (7%) ask about the addiction “sometimes”, and 1 (1%) did not ask at all. 39% of the respondents (*n* = 59) declared knowledge of the diagnostic and therapeutic treatment scheme in smokers. Answers to the open question about the methods used to assess the patient’s motivation to quit smoking were provided by 107 doctors, of whom 14 (13%) replied that they did not use any methods for assessment, 91 (85%) of the respondents indicated that they did it by “talking to the patient”, only 2 (2%) physicians perform the assessment using the dedicated questionnaire. The degree of smoking dependence among patients is assessed by 39 surveyed doctors (26%). 79 responders replied to the open question about the methods used to assess the degree of smoking dependence. Among the methods described, most doctors indicated asking about the number of cigarettes smoked a day and/or the duration of smoking. Only 4 people (5%) use specialized questionnaires for this purpose (the CAGE and Fagerström questionnaires were given). 130 surveyed doctors (86%) declared that they diagnose tobacco disease—ICD 10 F17 in patients addicted to smoking. Among the treatment methods recommended for patients addicted to smoking:

• 131 of the surveyed doctors (89%) stated “patients’ decision to quit smoking”.
• 68 (46%) recommend external anti-smoking counseling.
• 63 (43%) use pharmacological treatment.
• 51 (35%) indicated “support of the family”.

77 doctors (51%) answered the question about their experiences with methods of pharmacological cessation treatment, and among them 58 (75%) indicated nicotine replacement therapy, 25 (33%) used nicotinic receptor antagonists, and 17 (22%)—bupropion. 110 respondents (75%) showed interest in the training on tobacco cessation programs. The readiness to conduct smoking cessation therapy in their patients was declared only by 60 surveyed physicians (39%). Doctors without specialization and with seniority beneath 10 years showed greater willingness to educate themselves in the field of nicotine addiction therapies and to apply these methods in practice. The readiness to participate in the training was expressed by 85% of doctors without specialization and 68% of specialists. The distribution of responses regarding the willingness to deliver smoking cessation therapy was similar (56.41% and 33.62%, respectively). These relationships were statistically significant (*p* = 0.048 and *p* = 0.003). Similar results were obtained with comparison of the duration of work experience. A greater percentage of doctors working shorter than 10 years were willing to participate in training (88 vs. 64%; *p* = 0.003) and to perform smoking cessation therapy (56 vs. 21%; *p* = 0.001).

Among the answers to the open-ended question about the factors that could encourage doctors to perform routinely such therapy in their patients, the most frequently mentioned were: increasing the time for a patient’s visit, reimbursement from national health service, uniform recommendations for treatment, appropriate training, information about the effectiveness of the therapy, additional gratification, participation in a clinical trial (Table 4).

**Table 4:** The characteristic of surveyed doctor group with analysis of smoking status, declaration on readiness to participate in anti-smoking interventions training and to deliver smoking cessation therapy

	Total (%)	Smokers; *n* (%)	*p* value ^a^	Ready to participate in anti-smoking interventions training; *n* (%)	*p* value ^a^	Ready to deliver smoking cessation therapy; *n* (%)
Total	152 (100)	10 (6.58)		110 (72.37)		60 (39.47)
Specialist						
Yes	113 (74.34)	3 (2.65)	**0.003**	77 (68.14)	0.048	38 (33.62)
No	39 (25.66)	7 (17.95)		33 (84.62)		22 (56.41)
Sex						
Female	103 (67.76)	6 (5.83)	0.859	77 (74.76)	0.139	43 (41.75)
Male	49 (32.24)	4 (8.16)		33 (67.35)		17 (34.69)
Work experience						
< 10 lat	50 (32.89)	8 (16)	**0.004**	44 (88)	**0.003**	28 (56)
> 10 lat	100 (65.79)	2 (2)		64 (64)		31 (31)
Frequency of asking patients about smoking						
Sometimes	11 (7.24)	1 (9.09)	** < 0.001**	7 (63.64)	0.635	5 (45.45)
If disease is related to smoking	41 (26.97)	5 (12.2)		28 (68.29)		11 (26.83)
Always	99 (65.13)	3 (9.16)		75 (75.76)		44 (44.44)
Do not ask	1 (0.66)	1 (100)		0 (0)		0 (0)
Knowledge of diagnostic and therapeutic						
Management in smoking cessation therapy						
Yes	59 (38.82)	2 (3.39)	0.345	40 (67.8)	0.456	28 (47.45)
No	93 (61.18)	8 (8.6)		70 (75.27)		32 (34.41)
Assessing the degree of smoking dependence						
Yes	39 (25.66)	1 (2.56)	0.446	25 (64.1)	0.289	13 (33.33)
No	111 (73.03)	9 (8.11)		84 (75.68)		46 (41.44)
Recommend following treatment methods for smoking patients						
Patient’s decision to quit the addiction	130 (85.53)	8 (6.15)	0.918	97 (74.62)	0.490	54 (41.54)
Family support	51 (33.55)	0 (0)	0.046	41 (80.39)	0.257	21 (41.18)
Pharmacotherapy	62 (40.79)	5 (8.06)	0.793	49 (79.03)	0.316	27 (43.55)
External anti-smoking counseling	68 (44.74)	2 (2.94)	0.188	47 (69.12)	0.231	25 (36.76)
Type of performed work						
Public outpatient	102 (67.11)	5 (4.9)	0.380	78 (76.47)	0.112	39 (38.24)
Commercial outpatient	104 (68.42)	7 (6.7)	0.937	76 (73.08)	0.863	36 (34.62)
Surgery	97 (63.82)	6 (6.19)	0.772	77 (79.38)	0.039	43 (44.33)

Bold indicates *p* ≤ 0.05

## Discussion

No study has evaluated the impact of head and neck cancer diagnosis on the patient’s smoking status in Poland so far. Limited information addressing the smoking status of head and neck cancer patients after completed treatment is available. The other issue is understanding whether smoking cessation treatment strategies could be implemented in otolaryngology practice to effectively increase tobacco abstinence in this population. This pilot study was design to identify the needs of cancer patients for smoking counseling, dedicated personnel, certain types of smoking cessation intervention, possibilities of integration smoking cessation into ENT clinical flow for further analysis of potential model of tobacco treatment program in head and neck cancer patients, suitable for implementation in a cancer setting.

The evidence related to outcomes measure, comorbidities and quality of life support the issue of including smoking counseling to comprehensive treatment management for patients with cancer diagnosis [17]. Current guidelines from the American Association for Cancer Research and the National Institute for Clinical Excellence in United Kingdom recommended cessation assistance for all cancer patients [18, 19].

Correspondingly, patients with cancer diagnosis were identified as highly motivated group to stop smoking and most of them consider a quit attempt within 6 months of the diagnosis [20]. Similar opinions were expressed by 76% of patients participating in our study. Moreover, the results of Schneider motivation test confirmed adequate stimulus to quit smoking in 70% of them. The metanalysis by Nayan et al. revealed in patients with a diagnosis of head and neck cancer the perioperative period as a most important moment for successful smoking cessation interventions [21]. However, only 6 (12%) of surveyed patients declared the help of qualified personnel on previous smoking cessation attempts, although the median of the duration of disease symptoms was 8 months among the studied population. This may indicate low awareness among smokers of anti-smoking therapy and/or that such methods are not widely available. Considering that overall sustained smoking cessation rates are quite poor for head and neck cancer patients, the emphasizes should be on effective tobacco treatment programs in parallel with cancer therapy. Cinciripini et al. presented quite satisfactory results of smoking abstinence in cancer patients admitted to such tobacco treatment program implemented in oncologic setting with the 45.1% of quitters at 3 months, 45.8% at 6 months, and 43.7% at 9 months [22].

Currently, due to national campaigns addressing smokers even on cigarette packs, they are aware of major risks related to smoking, including the knowledge of its correlation to the cancer. However, tobacco use is a well-known addiction and although the assumption of its causative role, relapses post quitting smoking are quite frequent. de Almeida et al. constituted increased cessation rates among head and neck cancer patients with higher education, earlier stages of cancer disease, laryngeal cancer and surgery as a part of cancer treatment [23]. In our study, we found that patients with laryngeal and hypopharyngeal cancer show significantly lower scores of nicotine dependence comparing to those with oral cavity and oropharyngeal cancer. Moreover, the percentage of patients accepting smoking as a disease was significantly higher among those with lower nicotine dependence scores. The significantly higher nicotine dependence levels of patients with specific cancer localizations as well as higher scores of HSI among younger patients may indicate the need for enhanced follow-up and support in smoking counseling within these groups.

The literature exploring motivation to smoking cessation is quite extensive from developed countries and reveals comparable level among head and neck cancer patients from 70 to 80% [24–26]. Contrary, such research is scarce from countries with lower and middle income. We think that similar motivation to quitting smoke can be expected in other European countries, especially within the European Union, due to the comparable level of awareness among smokers on its harmfulness and common regulations on public health interventions. However, in other countries, there can exist specific barriers including cultural aspects, difference in health system and other stake-holding sectors, the interference of the tobacco industry in the governance politic that may influence patients’ awareness and motivation.

Apart from disadvantageous effect of smoking on cardio-vascular diseases and diabetes, relevant for consideration is the projected increase in cancer survivor’s population, including among them smokers, and therefore, the rise in rates of recurrences and second primary cancers. Integrating tobacco treatment in the oncologic setting can contribute to apparent financial benefits to ex-smokers and decrease in health care spending and resource use in the future [27]. Despite the evidence, standard oncological setting still has not incorporated smoking cessation programs in most cancer centres. Implementation of recommendations and guidelines is limited.

The critical appraisal of literature performed by Nasser prove effectiveness of behavioral intervention for smoking cessation by oral health professionals in reducing tobacco use in smokers and preventing relapses in quitters [28]. The author, however, was not able to give enough evidence for cost-effectiveness of such interventions or designate the most effective method. The same author confirms lack of reimbursement from the National Health System, lack of time and training and fears over patient response as main discourages for deliver smoking cessation activities by British oral health professionals [28]. The results of our survey confirm these observations among Polish ENT doctors, who in majority (61%) are not willing to conduct anti- smoking therapy in their patients and who give quite adequate propositions for encouragement to consider such therapy in comparison with British colleagues. The other reason for such restrained attitude among Polish doctors may be related to uncertainty about accurate management strategy. Since although majority of them declare investigation of smoking status among their patients, vast of them admitted that they are not familiar with diagnostic and treatment protocol for smoking cessation, and therefore, it was revealed that they do not use dedicated instruments for addiction and motivation assessment. Positive is the observation that quite large group of ENT doctors (75%) showed interest in the training on tobacco cessation programs, especially those with shorter work experience and without specialization. It may prove that ENT residents are more willing to acquire additional education in the field of nicotine addiction, and are also more motivated to use acquired skills and knowledge in medical practice.

The systemic review by McCarter et al. recommends an intensive, multicomponent approach for smoking head and neck cancer patients; however, they are unable to recommend any specific form of anti-smoking intervention because of only few well-designed prospective smoking cessation studies, and therefore, luck of evidence in this area [29].

Related to the topic, but revealing even more challenge, is the treatment approach for tobacco–alcohol-dependent patients. Acknowledged is that smoking rate positively correlate with severity of alcohol dependence, and severe alcohol dependency is related to higher nicotine dependence, and therefore, reduced odds of smoking cessation. To be effective in the treatment of both addictions, the question about proper sequence of the therapy (concurrent, sequential, or not linked at all) is risen by researchers, but there is still no consensus [30, 31].

Considering organization of effective cessation treatment program not only overcoming of limitations in funding and large patient volumes are the issue, but above all essential is enhancement of patient’s awareness of methods and the cancer-related benefits from quitting as well as integration of health care professional community to implement and sustain a comprehensive smoking cessation program in existing cancer care.

We recommend that each head and neck cancer smoking patient should be offered a smoking cessation treatment in oncological setting or should be referred for therapy to a professional smoking cessation center. Our results encourage both the development of a training program for otolaryngologists in tobacco counseling and a multicentre smoking cessation support project for that group of patients.

## Conclusions

Patients with diagnosis of precancerous lesions or cancer of upper aerodigestive track are highly motivated group to stop smoking and most of them consider a quit attempt due to the diagnosis. Relevant for smoking counseling programs are the facts that patients with oral cavity and oropharyngeal cancer show higher dependence to nicotine and that younger smoking patients present higher scores of Heaviness Smoking Index. Most ENT doctors are not willing to conduct anti-smoking therapy and they admit lack of knowledge of diagnostic and treatment protocol for smoking cessation, but most of them showed interest in the training on such programs.

## Data Availability

Data supporting reported results will be provided on reasonable request.
